# Modulation of Serum Brain-Derived Neurotrophic Factor by a Single Dose of Ayahuasca: Observation From a Randomized Controlled Trial

**DOI:** 10.3389/fpsyg.2019.01234

**Published:** 2019-06-04

**Authors:** Raíssa Nóbrega de Almeida, Ana Cecília de Menezes Galvão, Flávia Santos da Silva, Erick Allan dos Santos Silva, Fernanda Palhano-Fontes, João Paulo Maia-de-Oliveira, Lobão-Soares Barros de Araújo, Bruno Lobão-Soares, Nicole Leite Galvão-Coelho

**Affiliations:** ^1^Laboratory of Hormone Measurement, Department of Physiology and Behavior, Federal University of Rio Grande do Norte, Natal, Brazil; ^2^Postgraduate Program in Psychobiology, Department of Physiology and Behavior, Federal University of Rio Grande do Norte, Natal, Brazil; ^3^Onofre Lopes University Hospital, Federal University of Rio Grande do Norte, Natal, Brazil; ^4^Brain Institute, Federal University of Rio Grande do Norte, Natal, Brazil; ^5^National Science and Technology Institute for Translational Medicine (INCT-TM), Natal, Brazil; ^6^Department of Clinical Medicine, Federal University of Rio Grande do Norte, Natal, Brazil; ^7^Department of Biophysics and Pharmacology, Federal University of Rio Grande do Norte, Natal, Brazil

**Keywords:** ayahuasca, antidepressant, BDNF, biomarker, cortisol, depression, psychedelics, treatment-resistant

## Abstract

Serotonergic psychedelics are emerging as potential antidepressant therapeutic tools, as suggested in a recent randomized controlled trial with ayahuasca for treatment-resistant depression. Preclinical and clinical studies have suggested that serum brain-derived neurotrophic factor (BDNF) levels increase after treatment with serotoninergic antidepressants, but the exact role of BDNF as a biomarker for diagnostic and treatment of major depression is still poorly understood. Here we investigated serum BDNF levels in healthy controls (*N* = 45) and patients with treatment-resistant depression (*N* = 28) before (baseline) and 48 h after (D2) a single dose of ayahuasca or placebo. In our sample, baseline serum BDNF levels did not predict major depression and the clinical characteristics of the patients did not predict their BDNF levels. However, at baseline, serum cortisol was a predictor of serum BDNF levels, where lower levels of serum BDNF were detected in a subgroup of subjects with hypocortisolemia. Moreover, at baseline we found a negative correlation between BDNF and serum cortisol in volunteers with eucortisolemia. After treatment (D2) we observed higher BDNF levels in both patients and controls that ingested ayahuasca (*N* = 35) when compared to placebo (*N* = 34). Furthermore, at D2 just patients treated with ayahuasca (*N* = 14), and not with placebo (*N* = 14), presented a significant negative correlation between serum BDNF levels and depressive symptoms. This is the first double-blind randomized placebo-controlled clinical trial that explored the modulation of BDNF in response to a psychedelic in patients with depression. The results suggest a potential link between the observed antidepressant effects of ayahuasca and changes in serum BDNF, which contributes to the emerging view of using psychedelics as an antidepressant. This trial is registered at http://clinicaltrials.gov (NCT02914769).

## Introduction

There has been some recent debate about the efficacy of currently available antidepressants (Cipriani et al., 2018; Hengartner and Plöderl, 2018). Antidepressant drugs appear to act more efficiently than placebo mainly in severe major depression, while in patients with mild or moderate depression they have reduced benefit after drug treatment (Aherne et al., 2017). Moreover, the latency between administration and antidepressant response can take weeks (Strawn et al., 2018). Highlighting, however, that a large number of patients are undertreated, which results in pseudo-resistance to treatment (Sackeim, 2001). With careful augmentation of different antidepressants and other treatment modalities, about 9–10% of patients with depression have treatment-resistant depression (Ho and Zhang, 2016).

In this scenario, several research groups have intensified their search for new antidepressants, including psychedelic substances (Santos et al., 2016). Serotonergic psychedelics, from natural or synthetic origin, present a similar mechanism of action of current antidepressants, such as the agonism of serotonin (Sanchez, 2018). Therefore, there is an increasing number of studies testing the antidepressant effects of psychedelics in humans, particularly of psilocybin (Carhart-Harris et al., 2016; Griffiths et al., 2016; Ross et al., 2016) and ayahuasca (Osório et al., 2015; Sanches et al., 2016; Palhano-Fontes et al., 2018), including in animal models of depression (Silva et al., 2018). Moreover, recent studies have suggested that regular intake of micro-doses of psychedelics leads to benefits in mood, health and cognition, decreasing symptoms of depression and anxiety, with no reports of significant alteration of consciousness (Johnstad, 2018; Anderson et al., 2019; Polito and Stevenson, 2019).

Ayahuasca is a brew created by Amerindians whose antidepressant effects have been recently tested in an open label (Osório et al., 2015; Sanches et al., 2016) and a randomized controlled clinical trial (Palhano-Fontes et al., 2018) in treatment-resistant depression. The major components of ayahuasca, specifically *N*,*N*-dimethyltryptamine (*N*,*N*-DMT) and β-carbolines (tetrahydroharmine, harmine, and harmaline), act in several pathways involved in the etiology of depression (Carbonaro and Gatch, 2016). These β-carbolines are reversible monoamine oxidase inhibitors (Horák et al., 2018), tetrahydroharmine also acts as a serotonin reuptake inhibitor, while *N*,*N*-DMT is a serotonergic receptor 5-hydroxytryptamine 2A (5-HT_2A_) and sigma-1 receptor (σ1R) agonist (Carbonaro and Gatch, 2016).

Despite of the peculiarity of each psychedelic, it is known that several pathways of neuronal plasticity are activated by these compounds (Winkelman, 2017). The induction of neuroplasticity and neurogenesis by psychedelics are a relevant aspect of their action as potential antidepressants since several studies have shown that the efficacy of clinical antidepressants is related to an increase in hippocampal plasticity induced after monoaminergic enhancement (Dakic et al., 2016; Ardalan et al., 2017; Morales-García et al., 2017). On the other hand, a psycho-immune-neuroendocrine network alteration, which occur through the reduction of pro-inflammatory cytokines, has also been proposed to explain the effect of antidepressants (Lu et al., 2017; Stapelberg et al., 2018).

The activation of the 5-HT_2A_ and tyrosine kinase B receptor (TrkB) by some psychedelics induces neuroplasticity through rapamycin (mTOR) signaling pathway (Collo and Pich, 2018; Ly et al., 2018). On the other hand, the σ1R, which is involved in the modulation of several neurotransmitter systems, such as monoaminergic and glutamatergic pathways (Inserra, 2018), modulates neuroplasticity and neurogenesis by intracellular calcium-dependent signal (Tsai and Su, 2017). The elevation of glutamatergic activity in the prefrontal cortex by alpha-amino-3-hydroxy-methyl-5-4-isoxazolpropionic (AMPA) receptor increases in neuroplasticity by brain-derived neurotrophic factor (BDNF) (Vollenweider and Kometer, 2010; Marek, 2017; Yang et al., 2018). Moreover, cortisol, the main stress hormone, also modulates the BDNF expression, as well as the activity of BDNF receptor, TrkB, in central nervous system (Kunugi et al., 2010).

Recent *in vitro* studies have suggested that the β-carbolines present in ayahuasca induce proliferation, migration and differentiation of neurons (Morales-García et al., 2017) and that 5-methoxy-*N*,*N*-dimethyltryptamine prompts cascades involved in neuroplasticity (Dakic et al., 2017). Furthermore, it has been suggested that harmine increases BDNF in the hippocampus of rodent animal models after treatment (Fortunato et al., 2010; Brierley and Davidson, 2012). Taking these evidences into account, we hypothesize that BDNF plays a direct role in the neuroplasticity observed for different ayahuasca compounds.

Brain-derived neurotrophic factor is a neurotrophin synthesized by neurons and glial cells that has been strongly correlated with changes in volume of specific brain areas observed in patients with major depression (Foltran and Diaz, 2016). Patients with depression generally present neurodegeneration and smaller hippocampal and prefrontal cortex volume (Brown et al., 2018; Iimori et al., 2018; Roody et al., 2018) and such reductions are not only due to neuronal death, but also caused by decreased neuroplasticity and neurogenesis secondary reduced expression of neurotrophic factors, such as BDNF (Kraus et al., 2017; Jesulola et al., 2018). On the other hand, recent studies suggested increased expression of BDNF in some brain areas, such as the amygdala and the *nucleus accumbens* (Phillips, 2017).

In addition, preclinical and clinical evidence suggest that patients with depression tend to present lower levels of serum BDNF when compared to healthy individuals (Wilkinson et al., 2018; Ma et al., 2019), which may be reversed after antidepressant drug treatment (Brunoni et al., 2017; Molendijk et al., 2018). Baseline alterations in serum BDNF levels in depressive patients is not a consensus in the literature (Jevtović et al., 2011; Molendijk et al., 2011; Papakostas et al., 2011; Elfving et al., 2012) and changes in BDNF levels after treatment seem to depend on the antidepressant class (Kreinin et al., 2015; Sheldrick et al., 2017; Wilkinson et al., 2018). Nevertheless, novel antidepressants including agomelatine and vortioxetine were found to increase hippocampal BDNF level and of BDNF positive neurons in an animal model of depression (Lu et al., 2018a,b).

Therefore, this study investigated serum BDNF levels from a randomized placebo-controlled trial that tested the potential antidepressant effect of a single dose of ayahuasca in treatment-resistant depression (Palhano-Fontes et al., 2018). We expected that baseline serum BDNF levels will predict major depression, the BDNF levels should in turn depend on individual clinical characteristics, with patients with major depression disclosing lower baseline levels of serum BDNF when compared to healthy volunteers. Moreover, the BDNF levels should be correlated with serum cortisol. In addition, we hypothesize that volunteers treated with ayahuasca could present higher levels of serum BDNF than those treated with placebo and this BDNF levels would predict the improvements observed in depression severity.

## Materials and Methods

### Study Design

The study is a randomized double-blinded placebo-controlled trial using a parallel arm design (Palhano-Fontes et al., 2018), which was conducted at the psychiatry clinic at University Hospital Onofre Lopes (HUOL) of the Federal University of Rio Grande do Norte (UFRN). The study was approved by the Ethics Committee for Medical Research of the HUOL (#579.479) and was registered at http://clinicaltrials.gov (NCT02914769). The procedures of this study comply with the ethical standards of the relevant national and institutional committees for human experimentation and with the Declaration of Helsinki of 1975, revised in 2008. All subjects provided written informed consent prior to participation.

Seventy-three volunteers participated in the trial: a control group of healthy individuals (CG, *n* = 45; 20 men and 25 women) and a group of patients with treatment-resistant major depression (MD, *n* = 28; 7 men and 21 women). The greater presence of women than men in our sample reflects the real prevalence of this disorder in general population (American Psychiatric Association [APA], 2013; Kuehner, 2017). It is speculated that some biological and environmental characteristics can contribute to the observed gender difference (Vamvakopoulos and Chrousos, 1993; Panagiotakopoulos and Neigh, 2014). The MD group was composed by patients with major depression, exhibiting inadequate responses to at least two antidepressant medications from different classes. All patients were in a current moderate to severe depressive episode at baseline, based on the Hamilton Depression Scale (HAM-D) (Hamilton, 1960) and Montgomery–Åsberg Depression Rating Scale (MADRS) (Montgomery and Åsberg, 1979). HAM-D and MADRS are the most commonly used tool to assess depressive symptoms, and as a primary outcome of different clinical trials for depression (Rush et al., 2004; Howland, 2008). Before engaging in the study, patients were in wash-out period for 2 weeks, and therefore were not taking antidepressants during the study. The CG was formed by individuals without history or actual diagnosis of psychiatric or neurological disorders. All volunteers were at least 18 years old, were naïve to ayahuasca, with no previous experience with any other psychedelic substance and history of drug abuse or substance dependency. Pregnancy was also an exclusion criterion.

The volunteers were admitted to the hospital for a sleep-in 1 day before the dosing session (D-1), when the MADRS scale was applied. MADRS was also applied in the following day (D0; baseline), right before the dosing session. Participants were treated with either a single dose of 1ml/kg of ayahuasca (AYA) or placebo (PLA) in a 1:1 ratio. MADRS scores were again obtained 48 h (D2) after the dosing session. Blood samples for serum BDNF and serum cortisol quantification were collected at D0 and at D2. In order to avoid circadian oscillation, samples were collected always at 7:00 a.m. and volunteers were in 8 h of fasting.

Dosing sessions were placed in comfortable living room at HUOL, all protocol lasted approximately 8 h. After a light breakfast, around 800 a.m and before the start of the treatment (10:00 a.m), the volunteers were reminded about the effects of they could experience and were instructed with some strategies to help eventual difficulties. During dosing, which lasted for approximately 4 h, all volunteers were allowed to listen to a predefined music playlist and were tutored to maintain their eyes closed, while focusing on their body, thoughts and emotions. Moreover, two researchers remained in a room next door, offering assistance when needed. For more details on the methods used in the clinical trial, see Palhano-Fontes et al. (2018).

A single batch of ayahuasca was prepared by a branch of the Barquinha church, and used throughout the study, and its main alkaloids were quantified at two different instants along the period of the trial. The substance used as a placebo was a liquid intended to simulate the organoleptic properties of ayahuasca, besides giving rise to some nausea, vomiting and diarrhea, some of the most well-known effects of ayahuasca (Palhano-Fontes et al., 2018).

### Dosage of Molecules

Blood samples were analyzed in the Laboratory of Hormone Measurements (UFRN). Blood samples were centrifuged (10 min, 4°C at 3,000 rpm) and serum was distributed in aliquots of 0.300 μL and stored in freezers at −80°C. We used the CYT306 ELISA kit for BDNF dosage (Merck Millipore), which used a sandwich technique for measurement. For serum cortisol dosage we used the DGR-SLV 1887 ELISA kit that is a direct competitive ELISA. The absorbance was read using a spectrophotometer microplate reader (Epoch, Biotek Industries, Highland park, United States) with a Gen5 Data Analysis software interface.

### Statistical Analysis

Statistical analysis was conducted in SPSS 20. Graphics were built in R 3.4.1 (RStudio). The level of significance was set at *p* ≤ 0.05 (two-tailed).

#### Baseline Statements

All volunteers were categorized with respect to baseline levels of serum cortisol in three groups (Annane et al., 2006): hypocortisolemia (HC) (cortisol ≤ 15 μg/dL), eucortisolemia (EC) (15 μg/dL < cortisol < 43 μg/dL), and hypercortisolemia (cortisol > 44 μg/dL). We excluded individuals with hypercortisolemia due to insufficient sample size to statistical analyzes (MD = 0, CG = 4). BDNF and cortisol serum concentrations were converted to fit the logarithm scale.

A binary logistic regression was used to found the best model that predicts the presence of major depression in our sample. First, to investigate the directly modulation of BDNF on major depression, only the baseline serum BDNF levels was used as potential predictor (independent quantitative metrical variable) and the group (MD *n* = 28 and CG *n* = 45) as a dependent binary categorical variable. Second, the following potential predictors of depression were added in the model: sex (independent categorical variable), income (independent ordinal variable), age (independent quantitative metrical variable) and serum cortisol levels (EC and HC, independent categorical variable). These potential predictor variables were selected to analyses because: (i) current literature suggests a relation between depression, cortisol and BDNF; (ii) our sample has more women than men; and (iii) our sample of patients is older and has lower income than healthy controls.

A multi-linear regression was used to find the best model that predicts the baseline serum BDNF levels of all volunteers (*n* = 69; 27 men and 42 women). Here, the baseline serum BDNF was used as dependent quantitative metrical variable and the serum cortisol levels (EC and HC, independent categorical variable), sex (independent categorical variable), income (independent ordinal variable) and age (independent quantitative metrical variable) were used as potential predictor variables. These potential predictor variables were selected because: (i) current literature suggests a relationship between depression, cortisol and BDNF; (ii) our sample has more women than men and (iii) patients in our sample are older and have lower income than controls. Due to the sample size (*n* = 69), only models with a maximum of three output levels (predictor variables) were tested. The best model was decided according to Lowest Akaike’s Information Criteria (AIC), that is, when the model is statistically significant (*p* < 0.05), lower AIC values reflects the best model. However, if two or more models had the same AIC value, the result with the least number of predictors was considered the best model.

A multi-linear regression model was also used to test the potential modulation of clinical characteristics of patients on their baseline serum BNDF levels. Therefore, the baseline serum BDNF levels of patients were used as dependent quantitative metrical variable and the disease duration (in years; independent numerical variable), the index of disease duration by patient’s age (independent numerical variable), the number of previous episodes of depression (independent numerical variable), the number of previous unsuccessful antidepressant treatments (independent numerical variable), scores of MADRS of D-1 and D0 were used as predictor variables. The best model was decided according to Lowest Akaike’s Information Criteria. Due to the sample size (*n* = 28), only models with a maximum of two output levels (predictor variables) were tested.

After the binary logistic regression, the non-parametric Mann-Whitney test was used to analyze the possible statistical difference of the median of age (dependent quantitative metrical variable) between groups (MD and CG as independent categorical variable). In addition, the independent Student *T*-Test was used to analyze possible statistical difference of the media of baseline serum BDNF levels (dependent quantitative metrical variable) between cortisol levels (HC and EC as independent categorical variable).

Spearman correlation test was used to analyze the relationship between serum BDNF and serum cortisol for all volunteers.

#### After-Dosing Assessments

After the dosing session (D2), patients were classified with respect of treatment response, as remitters (R) or non-remitters (NR). Remission rates were defined as MADRS scores ≤10.

A General Linear Model (GLM) and the Fisher *post hoc* test were used to analyze the levels of serum BDNF between-group (MD and CG) and between-treatment (AYA and PLA) in each phase isolated (D0 or D2) and possible changes of serum BDNF levels before and after-treatment. The group and the type of treatment were used as independent categorical variables and serum BDNF levels at D0 and D2 were used as repeated dependent quantitative metrical variables, with residues adjustment. The outputs analyzed were: group, treatment, phase and the interaction between treatment^∗^phase, treatment^∗^group and phase^∗^group and across treatment^∗^group^∗^phase.

A binary logistic regression was used to find the best model that explained the remission rates at D2. Remission rate (R and NR) was used as a dependent binary categorical variable. The potential predictor variables tested were: the type of treatment (AYA or PLA, independent categorical variable), the number of previous unsuccessful antidepressant treatments (independent numerical variable), serum BDNF levels at D2 (dependent quantitative metrical variable) and sex (independent categorical variable).

Following binary logistic regression, the non-parametric Mann–Whitney test was used to analyze the possible statistical difference of the median of unsuccessful antidepressant treatments (independent numerical variable) in respect to the D2 remission rates (R and NR, as independent categorical variable).

## Results

### Baseline Assessments

Supplementary Figures S1, S2 show the consolidated standards of reporting trials (CONSORT) of this study for patients and controls, respectively.

All volunteers were Brazilians adults (MD: 41.57 ± 11.40 years, CG: 31.56 ± 9.90 years), most were women (MD: 75%, CG: 51.22%), most patients with secondary education (MD: 39.29%, CG: 34.15%) and CG with higher education (MD: 21.43%, CG: 56.10%). Most patients had lower income than controls (MD: 35.71%, CG: 12.19%). Most patients (57.14%) presented between 1 and 10 years of depression (28.57%: 11–17 years, 14.28%: 20–40 years). All patients were previously treated with at least 2 different antidepressants without remission: 75% used between 2 and 4 different drugs before and 25% of them used 5 or more. All patients (100%) had been treated already with a selective serotonin reuptake inhibitor (SSRI), 64.28% used tricyclic antidepressants (TCA), 53.57% serotonin–norepinephrine reuptake inhibitor (SNRI), and 25% had tried norepinephrine-dopamine reuptake inhibitor (NDRI) (Supplementary Figure S3). The percentage of antidepressants used does not add up to 100% because of the overlapping of previous antidepressants treatments. The socio-demographic and clinical characteristics are presented in Supplementary Table S1.

**FIGURE 1:**
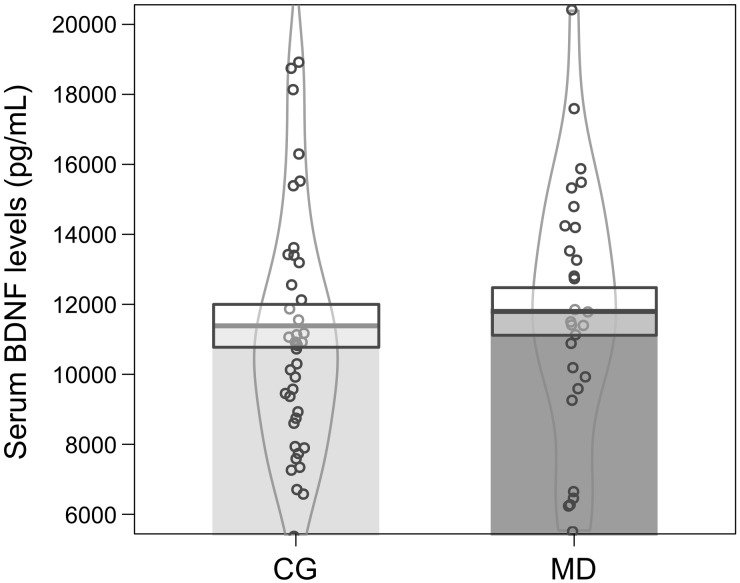
Mean ± standard error of serum BDNF levels (pg/mL) at baseline for patients (MD, dark gray color *n* = 28) and controls (CG, light gray color *n* = 41). The curvilinear gray lines represent the density of sample distribution. The circles represent individuals in each group.

**FIGURE 2:**
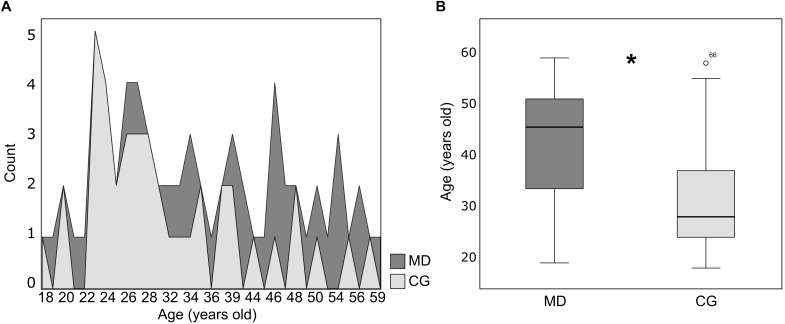
**(A)** Distribution of age (years old) for patients (MD, dark gray color *n* = 28) and healthy controls (CG, light gray color *n* = 41). **(B)** Box plot of age (years old) (median ± Q75% and Q25%) for the patients (MD, *n* = 28) and healthy controls (CG, *n* = 41). Mann–Whitney test, ^∗^*p* ≤ 0.05, statistically significant difference between-groups.

Baseline serum BDNF alone could not predict depression severity, as the model was not significant (Binary logistic regression: *X*^2^ = 0.222, df = 1, *p* = 0.63; *r*^2^ Nagelkerke = 0.004) (Figure 1). However, when the sex, income, age and cortisol level (EC and HC) were included as potential predictors, the model turned statistically significant (Binary logistic regression: *X*^2^ = 19.13, *df* = 5, *p* = 0.002; *r*^2^ Nagelkerke = 0.32). The model presents an explanation of 73.9% to predict depression severity, and age was the variable that best predicted the model (*B* = −0.06, df = 1, *p* = 0.02; 95% CI: 0.89; 0.99) (Figure 2A). In our sample, patients were older than the groups of healthy individuals (MD: 41.57 ± 11.40 years, CG: 31.56 ± 9.90 years) (Mann–Whitney: *U* = 302, *p* = 0.001) (Figure 2B).

Moreover, models were tested to find the best predictor of baseline serum BDNF levels for all volunteers (MD and CG). The best result, according to lowest Akaike’s Information Criteria, included only cortisol levels (EC and HC) as predictor with a low power of discrimination, where minor levels of cortisol predicts major serum BDNF levels of participants (multi-linear regression: AIC = −272.816, *B* = −0.081, *p* = 0.016; 95% CI: −0.147; −0.016). The results of all models tested as predictor of volunteers’ baseline serum BDNF are shown in Table 1.

**Table 1:** Potential predictor models of baseline serum BDNF levels of volunteers (multi-linear regression).

Potential predictor models	AIC values
**Cortisol levels**	**−272.816^∗^**
Sex	−268.934
Age	−268.93
Income	−268.93
Cortisol levels^∗^Sex	−272.816^∗^
Cortisol levels^∗^Age	−272.816^∗^
Cortisol levels^∗^Income	−272.816^∗^
Cortisol^∗^Sex^∗^Age	−272.816^∗^
Cortisol^∗^Sex^∗^Income	−272.816^∗^
Cortisol^∗^Income^∗^Age	−272.816^∗^

*^∗^Statistically significant model p ≤0.05. Bold text indicates the best model according to the Lower Akaike’s Information Criteria.*

**FIGURE 3:**
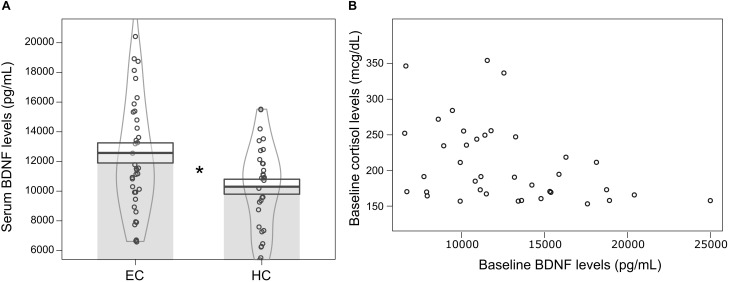
**(A)** Mean ± standard error of serum BDNF levels (pg/mL) at baseline for volunteers with hypocortisolemia (HC, *n* = 31) (serum cortisol < 15 mcg/dL) and eucortisolemia (EC, *n* = 38) (15 mcg/dL < serum cortisol < 43 mcg/dL). Independent Student *T*-test, ^∗^*p* ≤ 0.05, statistically significant difference between-groups. The curvilinear gray lines represent the density of samples distribution. The circles represent individuals in each group. **(B)** Significant negative correlation between serum BDNF and serum cortisol levels for eucortisolemic volunteers (MD and CG) at baseline (*N* = 38; Spearman test, *p* < 0.05, rho = –0.39).

Furthermore, we found that volunteers with hypocortisolemia (*n* = 31; μ = 10229.54 ± 506.92 pg/mL; 95% CI: 8992.79–11606.33) showed lower levels of baseline serum BDNF when compared to volunteers with eucortisolemia (*n* = 38; μ = 12574.61 ± 680.59 pg/mL; 95% CI: 11394.32–13754.91) (Independent Student *T*-test: *t* = −2,468, df = 67, *p* = 0.016) (Figure 3A). Moreover, volunteers with eucortisolemia showed a significant negative correlation between serum BDNF and serum cortisol (*n* = 38; *Spearman* test, *p* < 0.05, *rho* = −0.39) (Figure 3B). This correlation between BDNF and cortisol were not observed for the volunteers with hypocortisolemia (*n* = 31; *Spearman* test, *p* > 0.05, *rho* = −0.27).

Models using clinical characteristics of patients were tested to find potential predictors of their baseline serum BDNF levels, but none of those variables was statically significant when tested isolated or in models with two levels predictors. This result is detailed in Supplementary Table S2.

### After-Dosing Assessments

**FIGURE 4:**
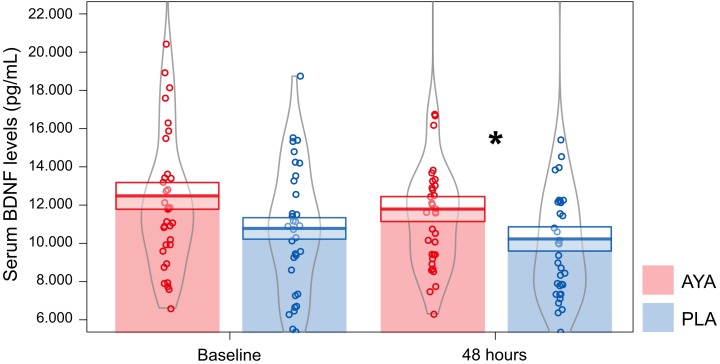
Mean ± standard error of serum BDNF levels (pg/mL) for volunteers of ayahuasca (AYA: red color, *n* = 35) and placebo (PLA: blue color, *n* = 34) treatments at baseline (D0) and 48 h after dosing (D2). GLM test and Fisher *post hoc*, ^∗^*p* ≤ 0.05, statistically significant difference between-groups. The curvilinear gray lines represent the density of samples distribution. The circles represent individuals in each group.

We found that volunteers treated with ayahuasca (*n* = 35; μ = 1179.81 ± 652.21 pg/mL; 95% CI: 10824.21–13149.12) had higher levels of BDNF at D2 than those treated with placebo (*n* = 34; μ = 10229.54 ± 633.58 pg/mL; 95% CI: 9452.28–11800.33) (GLM; Treatment: *F* = 4.81, df = 1, *p* = 0.03). The effect size (Cohen-*d*) between AYA and PLA at D2 was 0.53. At baseline we did not observe this difference between treatments (GLM; Treatment: *F* = 3.72, df = 1, *p* = 0.058). No difference in serum BDNF levels were observed between groups (DM and CG), phase (D0 and D2) and the respective interactions (Figure 4). Details of GLM statistical outputs are shown in Supplementary Table S3.

**FIGURE 5:**
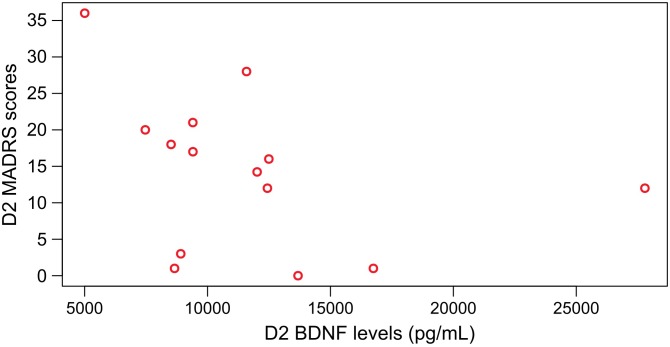
Significant negative Spearman correlation between BDNF levels and MADRS scores at D2 for patients that were treated with ayahuasca (*n* = 14; Spearman test, *p* ≤ 0.05 rho = –0.55).

Moreover, we found a negative correlation for patients treated with AYA between BDNF and MADRS at D2 (*n* = 14; *Spearman* test AYA, *p* ≤ 0.05, *rho* = −0.55) (Figure 5), which was not observed for patients treated with PLA (*n* = 14; *Spearman* test, PLA, *p* > 0.05, *rho* = −0.27).

Some variables were tested to find the best model that predicts remission rates at D2 (Supplementary Table S4 for statistical details). The analyses of these predictors isolated suggest that the number of previous unsuccessful antidepressant treatments was the best predictor of remission rates at D2 (Binary logistic regression *X*^2^ = 10.14, df = 1, *p* = 0.001; *r*^2^ Nagelkerke = 0.42) (*B* = −1.48, df = 1, *p* = 0.035; 95% CI: 0.075; 0.898). Previous treatment can predict D2 remission rates with power of explanation of 75% (Supplementary Table S4). Patients in remission at D2 (*n* = 11, μ = 2.9 ± 0.25) presented a smaller number of previous unsuccessful antidepressant treatments than patients that did not achieve remission (*n* = 17, μ = 4.5 ± 0.43) (Mann–Whitney: *U* = 33, *p* = 0.003).

We also explored other models with two possible predictors. For that, we tested the variable that was statistically significant in isolated models with others that were not significant (Supplementary Table S4). The best fit of these models used as parameters the number of previous unsuccessful antidepressant treatments and the type of treatment (AYA and PLA) (Binary logistic regression *X*^2^ = 10.764, df = 1, *p* = 0.005; *r*^2^ Nagelkerke = 0.43) (*B* = −1.34, df = 1, *p* = 0.04; 95% CI: 0.07; 0.97). However, in this model is the previous treatment that can predict D2 remission rate with 78.6% (Supplementary Table S4).

## Discussion

In this study, we found an interaction between serum BDNF and serum cortisol levels at baseline. The serum cortisol was predictor of baseline serum BDNF of volunteers. Individuals with hypocortisolemia, both patients and controls, presented lower serum BDNF levels when compared to volunteers with eucortisolemia (patients and controls). Moreover, we found a negative correlation between serum cortisol and serum BDNF in eucortisolemic volunteers. However, neither BDNF nor cortisol levels were predictors of major depression and none of the clinical characteristics of patients predicted their BDNF levels. Forty-eight hours after treatment (D2), we observed higher BDNF levels in both groups (patients and controls) who were treated with ayahuasca, compared to those treated with placebo. In addition, at D2 patients treated with ayahuasca, and not with placebo, presented a negative significant correlation between BDNF levels and MADRS scores. However, the number of previous unsuccessful antidepressant treatments was the main predictor of remission rates found at D2.

These results corroborate in part our initial hypotheses. On one hand, we found a relationship between serum cortisol and serum BDNF at baseline. Among the various responses induced by the glucocorticoid, the direct modulation of cortisol on BDNF appears to be extremely significant in understanding the etiology and treatment of major depression (Dai et al., 2018). The expression of BDNF in the central nervous system is modulated by various brain molecules, including cortisol. It is known that one interaction between these two molecules occurs at the level of messenger RNA (Tapia-Arancibia et al., 2004; Chou et al., 2016). It is observed that high concentrations of cortisol induce reduction of BDNF gene expression, and hippocampal and neocortex neurogenesis (Duman and Monteggia, 2006; Lupien et al., 2007; Ozan et al., 2010; Schwabe et al., 2012). Analogously, low concentrations of the cortisol interfere negatively in the expression of BDNF. An inverted U-curve relationship between cortisol and BDNF has been proposed, where intermediate levels of cortisol are the most suitable for the expression of BDNF (Tapia-Arancibia et al., 2004).

In addition, it is suggested that there is an interaction between TrkB and GR receptor activity. In order TrkB receptor to be activated by BDNF, the GR receptor must be coupled to the TrkB, forming a complex. When cortisol levels are high, cortisol binds to its GR receptor and removes it from the complex, reducing the potential response of BDNF (Numakawa et al., 2017). Therefore, the literature suggests that allostatic levels of cortisol are important to adequate BDNF expression and function. Herein, this hypothesis is corroborated by the model where baseline serum cortisol levels predict serum BDNF levels and by the negative correlation between BDNF and cortisol found only for eucortisolemic volunteers that consequently induced a baseline difference in serum BDNF levels of eucortisolemic and hypocortisolemic volunteers in our study.

Brain-derived neurotrophic factor is a broad-acting neurotrophin that plays important roles in protection, differentiation, viability and growth of new neurons (Lopes et al., 2017), and its effects go beyond the central nervous system (Wolkowitz et al., 2011; Pius-Sadowska and Machaliñski, 2017). Outside of nervous system, the BDNF is mainly synthesized in platelets, which also is the main storage site of peripheral tissues (Nurden, 2018). Rodent studies point out to the existence of bidirectional transport of BDNF in the blood–brain barrier (Khalin et al., 2016; Xing et al., 2016; Tharmaratnam et al., 2018), and peripheral administration of BDNF enhances hippocampal neurogenesis in mice, indicating that blood levels of BDNF are significant for brain function (Schmidt and Duman, 2010). Recent evidence suggests that several pathways of neuronal plasticity are activated by psychedelic compounds (Winkelman, 2017). The induction of neuroplasticity and neurogenesis by psychedelics are a relevant aspect of their action as potential antidepressants since several studies have shown that the efficacy of clinical antidepressants is related to an increase in hippocampal plasticity induced after monoaminergic enhancement (Dakic et al., 2016; Ardalan et al., 2017; Morales-García et al., 2017).

Even though most studies suggest that the serum BDNF levels of patients with depression are significantly lower than healthy controls (Gersner et al., 2014; Huang et al., 2017; Alves and Rocha, 2018; Wagner et al., 2018), this finding is not unanimous (Hellweg et al., 2008; Jevtović et al., 2011; Molendijk et al., 2011; Papakostas et al., 2011; Elfving et al., 2012). In our study, baseline serum BDNF levels, baseline serum cortisol levels, sex and income did not predict major depression and we did not find any clinical characteristics that predict baseline levels of BDNF in the group of patients. Both results are opposite to our hypothesis. Here, the variable age could predict the presence of depressive symptoms. However, as our patients were older than controls, this find might be biased by this baseline difference.

It has been speculated that wash-out periods may mask the expected reduced BDNF levels found in depression, as it is been proposed that antidepressants stimulate BDNF synthesis (Wilkinson et al., 2018). In fact, studies indicate that the reduction of BDNF is most consistently observed after a 4-week wash-out period (Brunoni et al., 2008; Yip et al., 2017) or in severe untreated patients (Kreinin et al., 2015). Herein, all patients were in a 2-week wash-out period, which might not have been enough to allow the detection of lower BDNF levels at baseline. Furthermore, there are still controversies regarding the correlation between blood and central BDNF levels. There is no consensus in the literature about how far human blood BDNF reflects accurately the concentration of BDNF in the central nervous system (Sen et al., 2008; MacQueen, 2009). Therefore, this could also explain why we did not find difference in BDNF levels between healthy controls and depressed patients and the baseline serum BDNF levels did not predict major depression in our study.

In addition, it is important to note that more recently three isoforms of BDNF have been identified. The intracellular synthesis of BDNF is initiated as the pre-pro-BDNF precursor form, and then the pre-region is removed resulting in the pro-BDNF form (Mizui et al., 2014; Foltran and Diaz, 2016), which after a new cleavage forms mature BDNF (m-BDNF) and BDNF pro-peptide. *In vitro* assays suggest that pro-BDNF is the major secreted form, not m-BDNF (Mowla et al., 2001; Kowiañski et al., 2017), but in the extracellular medium, the pro-BDNF may undergo cleavage by the action of plasmin (Diniz et al., 2018). The pro-BDNF/m-BDNF ratio seems to be critical for the determination of physiological or pathological conditions (Yoshida et al., 2012; Foltran and Diaz, 2016; Angoa-Perez et al., 2017). Increased pro-BDNF levels in the prefrontal cortex and ventral tegmental area, and reduced levels of m-BDNF in the prefrontal cortex and hippocampus are observed in different studies using animal models of depression (Lippmann et al., 2007; Shirayama et al., 2015; Hashimoto, 2016; Zhao G. et al., 2016; Zhao M. et al., 2016). Despite this, studies with humans that analyze pro-BDNF/m-BDNF ratio in blood are infrequent, and few studies showed changed ratio in patients with major depression (Yoshida et al., 2012; Zhou et al., 2013; Sodersten et al., 2014; Zhao et al., 2017). Both m-BDNF, BDNF pro-peptide and pro-BDNF can interact with Trk and p75^NTR^ receptor, although each one exhibits different biological activities (Mizui et al., 2014; Porcher et al., 2018). The m-BDNF has high affinity for TrkB receptors (Li et al., 2018) and its activation has been implicated in neural excitability and increased synaptic strength (Jiang et al., 2017). On the other hand, pro-BDNF and BDNF pro-peptide performs their biological actions mainly through the binding to p75NTR neurotrophin receptors and have been described as inhibitor of excitability and facilitators of synaptic depression (Mizui et al., 2014; Phillips, 2017). The commercial kit used in our study is not sensitive to different BDNF isoforms, and the measurement is related to the total BDNF amount of both isoforms.

We initially supposed that ayahuasca would modulate BDNF levels, and this was observed. After dosing (D2) we found higher levels of BDNF in individuals treated with ayahuasca compared to placebo, regardless of the group (MD or CG). The between-treatment effect size was medium. Additionally, patients that were treated with ayahuasca, and not with placebo, presented a negative significant correlation between BDNF levels and MADRS scores at D2: higher serum BDNF levels were correlated with lower depression symptomatology after the session with ayahuasca.

*N*, *N*-dimethyltryptamine (*N*,*N*-DMT), the main active compound of ayahuasca, activates 5-HT_2A_ and σ1R pathways involved in neuroplasticity (Fields and Mitchell, 2017; Inserra, 2018). Ayahuasca also induces an acute increase of cortisol levels (Santos et al., 2011; Galvão et al., 2018). Specifically, these same volunteers showed an increase of approximately 100% of salivary cortisol 1 h 40 min after ayahuasca intake, which probably was important to reach physiological levels of this hormone in hypocortisolemic volunteers (Galvão et al., 2018). Thus, the cortisol pathway could be critically implied in the mechanism related to how ayahuasca modulation of BDNF molecule. Although this study suggests higher levels of BDNF in volunteers treated with ayahuasca than those treated with placebo, a detailed explanation of specific pathways possibly involved in ayahuasca modulation of serum BDNF expression needs further pre-clinical and clinical investigation. Nevertheless, the early association between ayahuasca treatment and BDNF found herein 48 h after the dosing session gives support to a potential mechanism behind the observed antidepressant effects of ayahuasca. In this sense, it is also important highlight that previous studies also described significant increases in serum BDNF levels just after a longer period of antidepressant treatment, i.e., after 8–12 weeks (Gonul et al., 2003; Aydemir et al., 2005), only psychedelics with antidepressant potential that demonstrate rapid action via BDNF synthesis and secretion (Kavalali and Monteggia, 2015; Harmer et al., 2017).

However, at D2 neither serum BDNF levels nor the type of treatment (AYA or PLA) predicted remission rates at D2. It is important to highlight, however, that we did not observe a significant difference on remission rates at D2 between treatments (AYA and PLA) in our original clinical trial (Palhano-Fontes et al., 2018). In that study, the strongest effect size and a significant difference between treatments on response and remission rates were observed only at D7 (Palhano-Fontes et al., 2018), and we did not measure BDNF at D7. On the other hand, the number of previous antidepressant treatments predicted remission rates at D2, where patients that were in remission at D2 had a smaller number of previous unsuccessful antidepressant treatments than patients that did not achieve remission.

Despite previous findings indicating a positive correlation between antidepressant effects and the expression of BDNF (Zhou et al., 2017), not all studies support this finding (Castren and Rantamaki, 2008; Piccinni et al., 2008; Halaris et al., 2015). Few studies using double-blind placebo-controlled design have investigated BDNF in major depression (Teche et al., 2013; Brunoni et al., 2014), and studies with psychedelic substances evaluating depression patients are scarce. Ketamine, an anesthetic with psychedelic proprieties, have been tested in MD patients and animal models (Haile et al., 2014; Lepack et al., 2014; Allen et al., 2015; Ly et al., 2018), and patients with treatment resistant depression exhibited increased plasma BDNF after a ketamine session only in responders between 4 h after intake until 1 week later (Haile et al., 2014; Allen et al., 2015).

In addition to some limitations discussed above, other restrictions in the present study should be considered. First, the genetic factor related to BDNF was not considered in this work. Some studies suggest that major depressive disorder is directly linked to single nucleotide functional polymorphism of BDNF, leading to valine (Val) substitution by methionine (Met) at codon 66 (Val66Met), and heterozygous patients presenting the Val66Met polymorphism have more promising responses to antidepressants when compared to Val/Val homozygotes (Brandl and Walter, 2017; Kao et al., 2018). Second, serum cortisol and BDNF were measured only once, and considering that these molecules have circadian rhythm (Wright et al., 2015; Cain et al., 2017), studies that cover sequential measurements throughout the day and for different days could point to more precise results.

This was the first double-blind randomized placebo-controlled trial for depression that investigated changes in BDNF levels after an intervention with a psychedelic substance. The results point to important observations, such as the report of different baseline serum BDNF levels in respect to serum cortisol, higher levels of BDNF in volunteers treated with ayahuasca than placebo 48 h after the dosing session, as well a clear association between higher serum BDNF and lower symptoms of depression at D2. Our results suggest a potential link between the observed antidepressant effects of ayahuasca and changes in serum BDNF, which contributes to the emerging view of using psychedelics as an antidepressant.

## Ethics Statement

The study was approved by the Ethics Committee on Medical Research of HUOL by number 579.479 and was registered at http://clinicaltrials.gov (NCT02914769). The procedures of this work comply with the ethical standards of the relevant national and institutional committees for human experimentation and with the Declaration of Helsinki of 1975, revised in 2008.

## Author Contributions

NG-C, BL-S, DdA, JM-d-O, and FP-F designed the experiments. RdA and AG measured hormonal data. RdA, FP-F, DdA, ES, and AG collected experimental data, carried out statistical analysis, and prepared the figures. RdA, AG, FdS, NG-C, BL-S, DdA, and FP-F prepared the manuscript.

## Conflict of Interest Statement

The authors declare that the research was conducted in the absence of any commercial or financial relationships that could be construed as a potential conflict of interest.

## Abbreviations

AYA: ayahuasca
CG: control group of healthy individuals
D0: days of the dosing session
D-1: admitted on hospital
D2: 48 h after the dosing session
EC: eucortisolemia
HC: hypocortisolemia
MD: patients with treatment-resistant major depression
NR: non-remitted patients
PLA: placebo
R: remitted patients.
