# Supplementary material for: Modulation of Serum Brain-Derived Neurotrophic Factor by a Single Dose of Ayahuasca: Observation From a Randomized Controlled Trial
Source: Front Psychol. 2019 Jun 4;10:1234. doi: 10.3389/fpsyg.2019.01234 (PMC6558429; doi:10.3389/fpsyg.2019.01234)
Supplement: Supplementary file 1 [file Table_1.docx]

**Table S1.** The socio-demographic data of volunteers and clinical characteristics of patients

|  | **Sex** | **Age** | **Education** | **Employment status** | **Age at onset, y** | **Estimated illness duration, y** | **Number of previous episodes** | **Current episode, months** | **Past unsuccessful medications** | **Treatment classification** |
| --- | --- | --- | --- | --- | --- | --- | --- | --- | --- | --- |
|  |  |  |  |  |  |  |  |  |  |  |
| **DM1** | W | 36 | Inc. elem. education | Sick leave | 26 | 10 | 4 | 3 | **SSRI**(2), NDRI, SNRI | Aya |
| **DM2** | W | 54 | Inc. elem. education | Unemployed | 33 | 21 | 3 | 3 | **SSRI**(3), TCA | Aya |
| **DM3** | W | 34 | Undergraduate | Employed | 28 | 6 | 4 | 2 | **SSRI**(2), **NDRI** | Aya |
| **DM4** | M | 52 | Inc. elem. education | Unemployed | 38 | 14 | 2 | 2 | TCA(2), **SSRI**(2) | Aya |
| **DM5** | W | 56 | Secondary education | Unemployed | 53 | 3 | 1 | 36 | **SSRI**(2), **NDRI** | Aya |
| **DM6** | W | 47 | Postgraduate | Employed | 35 | 12 | 2 | 5 | **SSRI**(2), SNRI | Aya |
| **DM7** | M | 22 | Inc. undergraduate | Studying | 17 | 5 | 1 | 60 | SNRI(2), NDRI, SSRI, **TCA** | Aya |
| **DM8** | W | 45 | Inc. elem. education | Unemployed | 29 | 16 | 3 | 12 | TCA(2), **SSRI**(2) | Aya |
| **DM9** | M | 19 | Secondary education | Studying | 18 | 1 | 2 | 4 | **SSRI**, TCA | Aya |
| **DM10** | W | 39 | Secondary education | Unemployed | 38 | 1 | 2 | 6 | SSRI(2), **SARI**, TCA, **SNRI** | Aya |
| **DM11** | W | 47 | Postgraduate | Unemployed | 45 | 2 | 3 | 4 | **TCA**, **SSRI**(2) | Aya |
| **DM12** | W | 33 | Postgraduate | Employed | 21 | 12 | 2 | 12 | **SSRI**(3), SNRI(2), **TCA**, MA, **NDRI** | Aya |
| **DM13** | W | 32 | Inc. elem. education | Sick leave | 26 | 6 | 6 | 9 | TCA(2), SSRI(2)* | Aya |
| **DM14** | W | 40 | Elementary education | Unemployed | 26 | 14 | 3 | 48 | SSRI(2), **SNRI** | Aya |
| **DM15** | M | 27 | Secondary education | Employed | 21 | 6 | 2 | 1 | **TCA**(2), SSRI(2), NDRI, **SNRI** | Pla |
| **DM16** | W | 50 | Secondary education | Unemployed | 41 | 9 | 4 | 8 | **SSRI**(2), NSSRI | Aya |
| **DM17** | W | 52 | Postgraduate | Unemployed | 36 | 16 | 3 | 6 | **SNRI**, SSRI, TCA | Aya |
| **DM18** | M | 59 | Secondary education | Unemployed | 57 | 2 | 2 | 8 | SSRI(2), NDRI, SARI, **SNRI** | Pla |
| **DM19** | W | 34 | Inc. undergraduate | Unemployed | 9 | 25 | 8 | 14 | **SSRI**(2), **TCA**(4), SNRI(2), SARI | Pla |
| **DM20** | W | 49 | Inc. elem. education | Unemployed | 41 | 8 | 3 | 3 | **SSRI**(2), SNRI | Pla |
| **DM21** | W | 40 | Postgraduate | Employed | 38 | 2 | 2 | 6 | **SSRI**, TCA | Pla |
| **DM22** | W | 46 | Inc. elem. education | Unemployed | 13 | 33 | 5 | 36 | **SSRI**(4), SNRI, **TCA** | Pla |
| **DM23** | M | 21 | Inc. undergraduate | Employed | 19 | 2 | 1 | 24 | **SSRI**, **NSSRI** | Pla |
| **DM24** | M | 56 | Inc. elem. education | Unemployed | 17 | 39 | 5 | 9 | **SSRI**, **TCA**, SNRI | Aya |
| **DM25** | W | 26 | Secondary education | Sick leave | 24 | 2 | 3 | 6 | **SNRI**, TCA, SSRI | Aya |
| **DM26** | W | 54 | Elementary education | Unemployed | 40 | 14 | 4 | 12 | **SSRI**(2), TCA | Aya |
| **DM27** | W | 46 | Secondary education | Sick leave | 29 | 17 | 4 | 12 | **SSRI**(2), SNRI | Pla |
| **DM28** | W | 47 | Elementary education | Sick leave | 44 | 2 | 2 | 5 | **SSRI**(2), **TCA** | Pla |
| **C1** | W | 24 | Undergraduated | Unemployed | - | - | - | - | - | Aya |
| **C2** | M | 39 | Postgraduated | Autonomous | - | - | - | - | - | Aya |
| **C3** | M | 23 | Undergraduated | Employed | - | - | - | - | - | Aya |
| **C4** | W | 44 | Incomplete Undergraduated | Employed | - | - | - | - | - | Aya |
| **C5** | M | 26 | Secondary education | Employed | - | - | - | - | - | Aya |
| **C6** | W | 23 | Incomplete Undergraduated | Unemployed | - | - | - | - | - | Aya |
| **C7** | M | 20 | Incomplete Undergraduated | Unemployed | - | - | - | - | - | Aya |
| **C8** | M | 33 | Incomplete Secondary education | Employed | - | - | - | - | - | Aya |
| **C9** | W | 26 | Postgraduated | Employed | - | - | - | - | - | Aya |
| **C10** | W | 24 | Incomplete Postgraduated | Employed | - | - | - | - | - | Aya |
| **C11** | W | 18 | Incomplete Undergraduated | Employed | - | - | - | - | - | Aya |
| **C12** | W | 23 | Undergraduated | Unemployed | - | - | - | - | - | Aya |
| **C13** | W | 35 | Secondary education | Unemployed | - | - | - | - | - | Aya |
| **C14** | M | 23 | Incomplete Undergraduated | Unemployed | - | - | - | - | - | Aya |
| **C15** | M | 48 | Incomplete elementary education | Employed | - | - | - | - | - | Aya |
| **C16** | W | 48 | Secondary education | Employed | - | - | - | - | - | Aya |
| **C17** | M | 55 | Incomplete undergratuated | Employed | - | - | - | - | - | Aya |
| **C18** | W | 26 | Undergraduated | Autonomous | - | - | - | - | - | Aya |
| **C19** | M | 25 | Incomplete Undergraduated | Employed | - | - | - | - | - | Aya |
| **C20** | W | 39 | Postgraduated | Employed | - | - | - | - | - | Aya |
| **C21** | M | 46 | Postgraduated | Employed | - | - | - | - | - | Pla |
| **C22** | M | 28 | Undergraduated | Autonomous | - | - | - | - | - | Pla |
| **C23** | W | 35 | Postgraduate | Autonomous | - | - | - | - | - | Pla |
| **C24** | W | 24 | Undergraduated | Employed | - | - | - | - | - | Pla |
| **C25** | W | 31 | Postgraduated | Autonomous | - | - | - | - | - | Pla |
| **C26** | M | 37 | Postgraduated | Employed | - | - | - | - | - | Pla |
| **C27** | M | 20 | Incomplete undergraduated | Unemployed | - | - | - | - | - | Pla |
| **C28** | M | 25 | Incomplete Undergraduated | Nunca trabalhou | - | - | - | - | - | Pla |
| **C29** | M | 27 | Incomplete elementary education | Employed | - | - | - | - | - | Pla |
| **C30** | M | 23 | Incomplete undergraduated | Autonomous | - | - | - | - | - | Pla |
| **C31** | W | 28 | Incomplete elementary education | Employed | - | - | - | - | - | Pla |
| **C32** | W | 31 | Postgraduated | Employed | - | - | - | - | - | Pla |
| **C33** | M | 38 | Incomplete postgraduated | Employed | - | - | - | - | - | Pla |
| **C34** | W | 37 | Postgraduated | Employed | - | - | - | - | - | Pla |
| **C35** | M | 50 | Undergraduated | Unemployed | - | - | - | - | - | Pla |
| **C36** | W | 34 | Postgraduated | Employed | - | - | - | - | - | Pla |
| **C37** | W | 37 | Postgraduated | Employed | - | - | - | - | - | Pla |
| **C38** | W | 58 | Secondary education | Unemployed | - | - | - | - | - | Pla |
| **C39** | W | 24 | Postgraduated | Employed | - | - | - | - | - | Pla |
| **C40** | M | 32 | Postgraduated | Employed | - | - | - | - | - | Pla |
| **C41** | M | 27 | Incomplete postgraduated | Employed | - | - | - | - | - | Pla |

DM = Major Depression Group; C = Control Group; W=Woman; M=men; Inc=incomplete; Elem=elementary; TCA=tricyclic antidepressant; SSRI=selective serotonin-reuptake inhibitor; NDRI=noradrenaline–dopamine-reuptake inhibitor; NSSRI=noradrenaline and specific serotonin-reuptake inhibitor; SNRI=serotonin–noradrenaline reuptake inhibitor; SARI=serotonin antagonist and reuptake inhibitor; MA=melatonergic antidepressant. Current patients’ medication(s) before washout period appear in bold. *Patient A13 was not under treatment (for 6 months) at enrolment and did not remember the last medication used.
