# Supplementary material for: Modulation of Serum Brain-Derived Neurotrophic Factor by a Single Dose of Ayahuasca: Observation From a Randomized Controlled Trial
Source: Front Psychol. 2019 Jun 4;10:1234. doi: 10.3389/fpsyg.2019.01234 (PMC6558429; doi:10.3389/fpsyg.2019.01234)
Supplement: Supplementary file 2 [file Table_2.docx]

Table 2. Clinical characteristics as potential predictor models of baseline serum BDNF levels of patients with major depression resistant to treatment (Multi-linear regression p>0.05).

| Potential Predictor Models | AIC Values |
| --- | --- |
| Disease duration | -106.82 |
| Number of previous episodes of depression | -106.82 |
| Index of disease duration by patient’s age | -106.82 |
| Number of previous unsuccessful antidepressant treatments | -106.82 |
| MADRS of D0 | -106.82 |
| MADRS of D-1 | -106.82 |
| Disease duration*Number of previous episodes of depression | -106.82 |
| Disease duration*Index of disease duration by patient’s age | -106.82 |
| Disease duration* Number of previous unsuccessful antidepressant treatments | -106.82 |
| Disease duration* MADRS of D0 | -106.82 |
| Disease duration*MADRS of D-1 | -106.82 |
| Number of previous episodes of depression* Index of disease duration by patient’s age | -106.82 |
| Number of previous episodes of depression* Number of previous unsuccessful antidepressant treatments | -106.82 |
| Number of previous episodes of depression* MADRS of D0 | -106.82 |
| Number of previous episodes of depression*MADRS of D-1 | -106.82 |
| Index of disease duration by patient’s age *Number of previous unsuccessful antidepressant treatments | -106.82 |
| Index of disease duration by patient’s age * MADRS of D0 | -106.82 |
| Index of disease duration by patient’s age *MADRS of D-1 | -106.82 |
| MADRS of D0 *MADRS of D-1 | -106.82 |

Montgomery–Åsberg Depression Rating Scale (MADRS)
