# Supplementary material for: Modulation of Serum Brain-Derived Neurotrophic Factor by a Single Dose of Ayahuasca: Observation From a Randomized Controlled Trial
Source: Front Psychol. 2019 Jun 4;10:1234. doi: 10.3389/fpsyg.2019.01234 (PMC6558429; doi:10.3389/fpsyg.2019.01234)
Supplement: Supplementary file 3 [file Table_3.docx]

Table 3. Statistical values of main effects and interaction of General Linear Models (GLM) used for analyze of the changes of serum BDNF levels between baseline and 48h after dosing session (D2), for control group and patients with major depression of both treatments (ayahuasca and placebo).

| EFFECT | F | p | DF |
| --- | --- | --- | --- |
| Intercept | 75437.76 | 0.001 | 1 |
| Group | 1.63 | 0.20 | 1 |
| Treatment | 4.81 | 0.03 | **1** |
| Phase | 2.22 | 0.14 | 1 |
| Group*Treatment | 0.08 | 0.77 | 1 |
| Group*Phase | 0.42 | 0.51 | 1 |
| Treatment*Phase | 0.08 | 0.76 | 1 |
| Group*Treatment*Phase | 3.90 | 0.053 | 1 |
| Error |  |  |  |

^All values in black correspond to statistical significance and values in gray to non-significant ones.^
