# Supplementary material for: Modulation of Serum Brain-Derived Neurotrophic Factor by a Single Dose of Ayahuasca: Observation From a Randomized Controlled Trial
Source: Front Psychol. 2019 Jun 4;10:1234. doi: 10.3389/fpsyg.2019.01234 (PMC6558429; doi:10.3389/fpsyg.2019.01234)
Supplement: Supplementary file 4 [file Table_4.docx]

Table 4. Potential predictor models of remission rates at D2 (Binary logistic regression).

| Model | Predictor variable | Remission D2 | % explanation |
| --- | --- | --- | --- |
| MODEL 1 | Type of treatment | X² = 1.36 p= 0.243 | - |
| MODEL 2*  X² = 10.14 p= 0.001 | **Number of previous unsuccessful antidepressant treatments** | **B = -1.48 df =1 p = 0.035** | **75%** |
| MODEL 3 | D2 serum BDNF levels | X² = 1.32 df = 1 p 0.25 | - |
| MODEL 4 | Sex | X² = 0.05 p= 0.824 | - |
| MODEL 5*  X² = 10.76 df = 2 p = 0.005 | **Number of previous unsuccessful antidepressant treatments***  **Type of treatment** | **B = - 1.34 df = 1 p = 0.04**  **B = -0.75 df = 1 p = 0.43** | **78.6%** |
| MODEL 6*  X² = 10.14 df = 2 p =0.006 | Number of previous unsuccessful antidepressant treatments*  D2 serum BDNF levels | B = -0.47 df = 1 p = 0.98  B = -1.48 df = 1 p =0.04 | 75% |
| MODEL 7*  X² = 10.39 df = 2 p= 0.006 | Number of previous unsuccessful antidepressant treatments*  Sex | B = - 1.52 df = 1 p = 0.03  B = 0.62 df = 1 p = 0.62 | 75% |

* ^Statistically significant model p< 0.05^

^Bold text indicates the best models to explain the remission rate of D2 at each output level (one or two predictor).^
