# Supplementary material for: Modulation of Serum Brain-Derived Neurotrophic Factor by a Single Dose of Ayahuasca: Observation From a Randomized Controlled Trial
Source: Front Psychol. 2019 Jun 4;10:1234. doi: 10.3389/fpsyg.2019.01234 (PMC6558429; doi:10.3389/fpsyg.2019.01234)
Supplement: Supplementary file 5 [file Data_Sheet_1.docx]

Supplementary Material

**Figure 1**

**CONSORT: MAJOR DEPRESSION GROUP**

# Allocation

# Patients

Analysed (n = 14)

Analysed (n = 14)

Allocated to placebo (n = 18)

Received allocated intervention (n = 14)

Did not receive allocated intervention (n = 4)

- Didn’t meet criteria for depression after wash-out (n = 3)
- Insufficient sample (n = 1)

Allocated to ayahuasca (n = 17)

Received allocated intervention (n = 14)

Did not receive allocated intervention (n = 3)

- Dropped out (n = 1)
- Didn’t meet criteria for depression after wash-out (n = 2)

**Analysis**

**Patients**

Randomized (n = 35)

Excluded (n = 183)

- Not meeting inclusion criteria (n = 143)
- Refused to participate (n = 26)
- Other reason (n = 14)

Assessed for eligibility

(n = 218)

# Enrollment

# Patients

Flow diagram of patients group of the double-blind parallel randomized clinical trial

**Figure 2**

**CONSORT: CONTROL GROUP**

Analysed (n = 21)

Excluded after analysis

- Hypercortisolemia (n = 2)

Analysed (n = 20)

Excluded after analysis

- Hypercortisolemia (n = 2)

**Analysis**

**Patients**

Excluded (n = 61)

- Excluded pre-screening (n = 50)
- Not meeting inclusion criteria (n = 4)
- Refused to participate (n = 7)

# Allocation

# Patients

Assessed for eligibility

(n = 110)

Allocated to placebo (n = 24)

Received allocated intervention (n = 22)

Did not receive allocated intervention (n = 2)

- Dropped out

Allocated to ayahuasca (n = 25)

Received allocated intervention (n = 23)

Did not receive allocated intervention (n = 2)

- Dropped out

# Enrollment

# Patients

Randomized (n = 49)

Flow diagram of control group of the double-blind parallel randomized clinical trial.

**Figure 3**

**
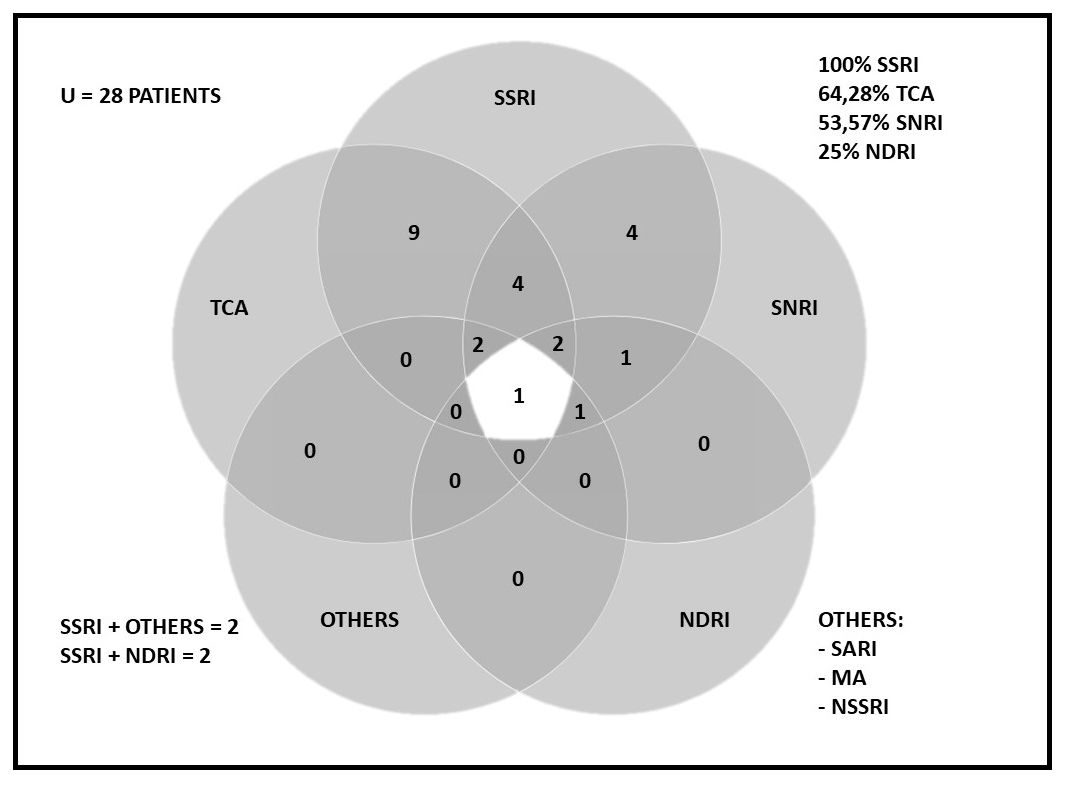
**

Venn diagram of previous antidepressants used by patients. TCA=tricyclic antidepressant; SSRI=selective serotonin-reuptake inhibitor; NDRI=noradrenaline–dopamine-reuptake inhibitor; NSSRI=noradrenaline and specific serotonin-reuptake inhibitor; SNRI=serotonin–noradrenaline reuptake inhibitor; SARI=serotonin antagonist and reuptake inhibitor; MA=melatonergic antidepressant.
